# Circadian Mechanisms of Food Anticipatory Rhythms in Rats Fed Once or Twice Daily: Clock Gene and Endocrine Correlates

**DOI:** 10.1371/journal.pone.0112451

**Published:** 2014-12-11

**Authors:** Danica F. Patton, Ângela M. Katsuyama, Ilya Pavlovski, Mateusz Michalik, Zachary Patterson, Maksim Parfyonov, Andrea N. Smit, Elliott G. Marchant, John Chung, Alfonso Abizaid, Kai-Florian Storch, Horacio de la Iglesia, Ralph E. Mistlberger

**Affiliations:** 1 Department of Psychology, Simon Fraser University, Burnaby, BC, Canada; 2 Department of Biology, University of Washington, Seattle, WA, United States of America; 3 Department of Neuroscience, Carleton University, Ottawa, ON, Canada; 4 Department of Psychiatry, McGill University, Montreal, PQ, Canada; Kent State University, United States of America

## Abstract

Circadian clocks in many brain regions and peripheral tissues are entrained by the daily rhythm of food intake. Clocks in one or more of these locations generate a daily rhythm of locomotor activity that anticipates a regular mealtime. Rats and mice can also anticipate two daily meals. Whether this involves 1 or 2 circadian clocks is unknown. To gain insight into how the circadian system adjusts to 2 daily mealtimes, male rats in a 12∶12 light-dark cycle were fed a 2 h meal either 4 h after lights-on or 4 h after lights-off, or a 1 h meal at both times. After 30 days, brain, blood, adrenal and stomach tissue were collected at 6 time points. Multiple clock genes from adrenals and stomachs were assayed by RT-PCR. Blood was assayed for corticosterone and ghrelin. *Bmal1* expression was quantified in 14 brain regions by in situ hybridization. Clock gene rhythms in adrenal and stomach from day-fed rats oscillated in antiphase with the rhythms in night-fed rats, and at an intermediate phase in rats fed twice daily. Corticosterone and ghrelin in 1-meal rats peaked at or prior to the expected mealtime. In 2-meal rats, corticosterone peaked only prior the nighttime meal, while ghrelin peaked prior to the daytime meal and then remained elevated. The olfactory bulb, nucleus accumbens, dorsal striatum, cerebellum and arcuate nucleus exhibited significant daily rhythms of *Bmal1* in the night-fed groups that were approximately in antiphase in the day-fed groups, and at intermediate levels (arrhythmic) in rats anticipating 2 daily meals. The dissociations between anticipatory activity and the peripheral clocks and hormones in rats anticipating 2 daily meals argue against a role for these signals in the timing of behavioral rhythms. The absence of rhythmicity at the tissue level in brain regions from rats anticipating 2 daily meals support behavioral evidence that circadian clock cells in these tissues may reorganize into two populations coupled to different meals.

## Introduction

Behavior and physiology in mammals are regulated by a multi-oscillatory circadian timekeeping system that is synchronized (entrained) to local time by sensitivity to environmental time cues (‘Zeitgebers’). Daily light-dark (LD) cycles are typically viewed as the most powerful zeitgeber, as these dominate phase control of circadian oscillators in the suprachiasmatic nucleus (SCN), a master clock that regulates the timing of oscillators elsewhere in the brain and body, via neural, endocrine and behavioral pathways [1], [2], [3]. Paramount among the behavioral pathways is the daily rhythm of food intake, which is normally synchronous with the daily sleep-wake rhythm. However, if food is restricted to the usual sleep phase, corresponding to the light period in nocturnal rats and mice, circadian clocks in peripheral organs and in many brain regions exhibit a marked shift in phase, as do physiological functions associated with these tissues [4], [5]. In parallel with shifted physiological functions, a behavioral rhythm of activity emerges that anticipates the mealtime [6], [7], [8]. The circadian system in mammals therefore exhibits a remarkable adaptive plasticity, enabling animals to concentrate foraging efforts at times of day when food is most likely to be found, and to adjust physiological and metabolic rhythms to optimize the absorption, utilization and storage of ingested nutrients. Despite the profound alteration of circadian organization in food-restricted mammals, the phase of the master SCN pacemaker, relative to the LD cycle, has most often been observed to remain unperturbed [9], [10]. In addition, circadian clocks and physiology in rats and mice sustaining complete ablation of the SCN entrain robustly to a daily mealtime [6], [7], [8]. Thus, although LD cycles determine the timing of circadian rhythms when food is available ad-libitum, food intake is the proximate zeitgeber for most circadian oscillators outside of the SCN pacemaker, and dominates when schedules of food availability and LD are in conflict.

The rhythm of food anticipatory behavioral activity that is induced by scheduled feeding exhibits canonical properties of circadian clock control, and has thus been modeled as the output of food-entrainable circadian oscillators (FEOs) [6], [7], [8]. Despite considerable effort, the location of these oscillators has not been definitively established; numerous brain regions and peripheral signals have been assessed for a role in food anticipatory rhythms, but none so far have been identified as essential [11], [12]. Mammalian circadian rhythms emerge from cell-autonomous transcription-translation feedback loops, and the clock genes that comprise these loops exhibit food-entrained daily rhythms of expression in many brain regions [13], [14]. It is thus conceivable that food anticipatory behavioral rhythms are regulated by a population of FEOs that are anatomically distributed, and potentially diverse in the meal-associated stimuli to which they entrain.

Further complicating the analysis of food-entrainable behavioral rhythms is the evidence that food restricted rats and mice can robustly anticipate two daily meals, in the day or the night [15], [16]. Notably, the two bouts of activity in rats can be dissociated under various experimental conditions, in a manner suggesting mediation by the uncoupling and separate entrainment of two FEOs [17], [18], [19], [20]. Whether this reflects uncoupling of oscillators within one structure, bilaterally, or in different structures is unknown. Dissociation of two bouts of food anticipatory activity can also be modeled by a single FEO, using the concept of a continuously consulted clock. The defining attribute of a continuously consulted clock is that it can emit representations of circadian phase that can be discriminated and attached to environmental events such as meals, permitting recognition and memory for mealtime. A FEO that can also be used as a consulted clock could potentially trigger bouts of food anticipation to multiple daily meals. Some cognitive rules would also need to be invoked, to explain anticipation of meals provided at different periodicities, e.g., once every 24 h and once every 24.5 h [17], [18], [19]. The requirement for complex rule learning to account for anticipation of two daily meals using one FEO sacrifices the advantage in parsimony that would normally fall to a one oscillator model.

Behavioral evidence has firmly established the ability of rodents to anticipate at least two daily meals, but has not produced evidence sufficient to reject either the single oscillator or the multiple oscillator models. Ultimately, circadian oscillators will have to be tracked in brain and peripheral tissues, to determine if anticipation of two daily meals is associated with unimodal or bimodal rhythmicity in these tissues. A single consulted clock model would predict that rats fed twice daily at 12 h intervals, e.g., once in the light period and once in the dark period, would exhibit unimodal rhythms of clock gene expression in rhythmic tissues, with these rhythms aligned to the nighttime meal (requiring little or no phase shift), the daytime meal (requiring a large shift), or stabilizing at a compromise phase. By contrast, a dual FEO model would predict bimodal rhythms in at least one clock gene expressing region, if clock cells entrained to different meals are topographically segregated, and would predict absence of rhythms at the tissue level, with intermediate levels of expression, if clock cells entrained to different meals are interspersed within a tissue.

As a first approach to this problem, we examined the expression of the circadian clock gene *Bmal1* in multiple brain regions, and the clock genes *Per1* and *Per2* in a subset of these regions, at 6 times of day, in rats anticipating a nighttime meal, a daytime meal or a half meal at each of these times. We chose regions of interest based on prior reports that these areas expressed circadian rhythms of clock genes that were reset by daytime feeding schedules in rats or mice. Although behavioral food anticipation rhythms persist in mice lacking *Bmal1* [20], [21], [22], the anticipation rhythm loses a canonical circadian clock property, namely, its circadian limits to entrainment [23]. Furthermore, food anticipation rhythms to daytime feeding are virtually absent in a *Per2^brdm^* mutant mouse [24], and the periodicity of oscillators driving food anticipation are markedly shortened in triple knockout mice lacking *Per1*, *Per2* and *Per3* [25]. Thus, even if other genes or non-transcriptional, biochemically based circadian oscillators [26] are involved in generating food-entrainable rhythms at the cellular level, the known clock genes *Bmal1, Per1* and *Per2* are clearly also important and retain value as cellular markers of entrainment.

In addition to clock genes in the brain, we also examined clock gene rhythms in two peripheral tissues (the stomach and the adrenal gland), as well as rhythms of hormones released by these tissues (gastric ghrelin and adrenal corticosterone). Peripheral oscillators and clock driven hormones have been proposed to provide time signals that drive physiological and possibly behavioral anticipatory rhythms in rodents anticipating one daily meal [27], [28], [29] but how these signals are affected by multiple meal schedules has not been thoroughly evaluated, and may provide novel insights into the potential roles that these clocks and their outputs play in the induction of behavioral rhythms.

## Methods

### Animals and Procedure

Young male Sprague Dawley rats (N = 108, 150–175 g on arrival) were housed in standard polypropylene cages with passive infrared motion sensors mounted over the cages, in cabinets with a 12∶12 LD cycle (∼30 lux during the light phase: <1 lux red, during the dark phase). Motion sensors were monitored continuously with the Clocklab data acquisition system (Actimetrics, Wilmette, IL, USA). The rats were allowed 12 days to entrain to the LD cycle, during which time standard rat chow (Bio-Serve, Frenchtown, NJ, USA) was provided ad libitum and average daily intake was recorded. All protocols were approved by the Animal Care Committee at Simon Fraser University (protocol 935p-09).

### Restricted Feeding Procedure

On day 13 the rats were randomly assigned to 1 of 3 restricted feeding schedules (36 rats per group). Rats in the ‘day-fed’ group received food for 2 h/day beginning 4 h after lights on (Zeitgeber Time 4, where ZT0 is lights-on, by convention). Rats in the ‘night-fed’ group received food for 2 h/day beginning 4 h after lights-off (ZT16). Rats in the ‘2-meal’ group received food for 1 h twice daily, at both ZT4 and ZT16. In the 1-meal schedules, each meal consisted of 65% (13.0±0.5 g) of the average total daily intake, a degree of caloric restriction that rats tolerate well (e.g., enhances longevity), and that induces robust food anticipatory activity. In the 2-meal schedule, half of that amount was provided at each mealtime. This is an amount per meal that is high enough to induce robust food anticipatory activity, and low enough to ensure that the rats eat all or most of the food at each meal, each day. On day 33 of the feeding schedules the lights remained off and no food was provided. The feeding and LD schedules resumed on days 34-36, and blood and tissue collection began on day 37.

### Blood and Tissue Collection

Beginning at ZT0 on day 37, rats were euthanized via CO_2_ in groups of 6 (2 or 3 rats/feeding schedule) at 6 time points (ZT0, 4, 8, 12, 16, 20). Tissue collection (euthanasia) times within each of the three feeding schedule groups began 20 h after the last meal (see S1 Figure for sampling times and number of hours since last meal). Blood samples for ghrelin and corticosterone assays were collected by cardiac puncture. The stomach and adrenals were then removed and frozen on dry ice. Upon decapitation, brains were rapidly removed and placed into methyl butane kept between −30 to −35° for 5 min. The brains were then transferred to and covered with powdered dry ice for 5 min. A thin layer of embedding matrix was applied to the ventral surface of the brain. The brains were then wrapped in autoclaved tin foil and placed in a −80°C freezer until sectioned.

### In situ hybridization

Brains were sectioned at 15 µm using a freezing cryostat (Thermo Scientific). The sections were divided into 8 series onto vectabond (Vector Labs) treated slides, made according to the manufacturers instructions. Slides were kept at −20°C during sectioning, and then transferred to dry ice and placed back in the −80°C freezer until processed using in situ hybridization.

Prehybridization: The slides were briefly fixed for 5 min in 4% paraformaldehyde in 0.1 M phosphate buffer (PB). The slides were then rinsed twice in 2 x SSC buffer (150 mM NaCl, 15 mM sodium citrate, pH = 7.0) and acetylated with 0.25% acetic anhydride in TEA HCl solution (100 mM Triethanolamine, 154 mM NaCl, pH = 8.0) for 10 min. Slides were quickly rinsed in 2 x SSC buffer and dehydrated as follows (all ethanols were prepared in DEPC-treated water): 1 min in 70% ethanol, 1 min in 80% ethanol, 2 min in 95% ethanol, 1 min in 100% ethanol, 5 min in chloroform, 1 min in 100% ethanol and then 1 min in 95% ethanol. Slides were then air dried in preparation for application of the pre-hybridization buffer (50% deionized formamide and 50% 4 x SSC). Each slide was cover-slipped with 50 µl of pre-hybridization buffer and incubated for 1 h at 37°C. The slides where then re-covered using 35 µl of hybridization buffer containing a 35S-labelled riboprobe transcribed from pGEM-T Easy Vector containing the following cDNA inserts: 981-bp rat *Per1*, 760-bp rat *Per2* or 602-bp mouse *Bmal1* (96% identity to rat *Bmal1*). The slides were incubated overnight at 55°C.

Post Hybridization: Slides were washed in a series of 1 x SSC baths (3 quick rinses and then two 10 min baths). Slides were then bathed twice in 50% formamide/50% 4 x SSC at 52°C for 5 and 20 min each. The slides were then quickly rinsed in 2 x SSC at room temperature and incubated in RNAse buffer (500 mM NaCl, 1 mM EDTA, 1 mM Tris pH = 8.0) containing RNAse A for 30 min at 37°C. Slides were again washed in 50% formamide/50% 4 x SSC at 52°C for 5 min and subsequently dehydrated as follows: 70% ethanol in 0.1 x SSC for 3 min, 80% ethanol in 0.1XSSC for 3 min, 95% ethanol in 0.1 x SSC for 3 min, a brief ddH_2_0 wash, 70% ethanol in water for 3 min and then air-dried. Slides where then exposed to autoradiographic films. Films were then scanned in a double-bedded, transparency scanner at 3600 dpi. Scans were then converted to jpegs and optical densities (OD) were calculated using NIH Image J. At least two brain sections were processed for each region of interest (S2 Figure). The average OD was measured for the area of interest and normalized to the average background OD for that slice [30]. During tracing of areas of interest, the tissue from rats in the two meal group was examined carefully for possible bilateral asymmetry of *Bmal1*, *per1 or per2* gene expression. No asymmetries were evident and data were therefore analyzed unilaterally.

### Corticosterone Assays

Cardiac samples were rapidly cooled and then stored at 4°C overnight. Samples were then centrifuged at 4000 rpm for 10 min after which the serum was collected and frozen at −20°C until analysis. Serum levels of corticosterone were determined using a commercially available radio-immunoassay kit (MP Biomedicals, Orangeburg, NY, USA).

### Ghrelin Assays

Cardiac blood samples (1 mL) were added to prepared tubes containing both EDTA and 100 µl/ml of apoprotenin (Phoenix Pharmaceuticals Inc, Burlingame, CA, USA) and stored on crushed ice for 45 min. Samples were then centrifuged for 15 min and plasma was collected into DNAse/RNAse-free tubes. 7 µl/100 µl of HCL was added to the plasma to decrease the pH to 4.0. Samples were then transferred to dry ice and subsequently stored in a −80°C freezer until analysis. Active ghrelin levels in plasma were determined using a Rat/Mouse Ghrelin (active) ELISA kit (Millipore, Billerica, MA, USA)

### Quantitative RT PCR

Stomach and adrenal samples were analyzed using quantitative RT-PCR for *Bmal1, Npas2, Rev-erb Per1, Per2*, and *Gapdh* (for primers see S1 Table). Samples were homogenized using a polytron or motor and pestle, and total RNA was isolated using TRIzol reagent (Invitrogen, Burlington, ON, CA). Total RNA samples were then adjusted to 50 µg/µl and cDNA was prepared from 10 µl (500 ng/µl) of RNA using the high capacity cDNA reverse transcription kit with RNAse inhibitor (Applied Biosystems, Burlington, ON, CA). cDNA samples (2 µl per reaction) were then run in triplicate with SYBR Green (20 µl per reaction) Applied Biosystems, Burlington, ON, CA) on a real time PCR detection system (Biorad).

### Data Analysis

Activity data were displayed visually in the form of actograms (plotted using Circadia, Dr. Tom Houpt legacy software, Florida State University) and average waveforms (plotted using GraphPad Prism 6, La Jolla CA) to confirm the presence of anticipatory locomotor activity prior to daily mealtimes. For hormone and ISH OD data, sample sizes of 6 per time point (N = 6 time points) per restricted feeding condition (N = 3 schedules) were planned, but variability in hormone assays or brain sectioning resulted in smaller sample sizes for one or more groups at one or more time points. Sample sizes for RT-PCR data were set at 3 per time point per feeding group (half of the samples were analyzed, randomly selected). The significance, phase and amplitude of 24 h rhythms in hormone and clock gene expression were evaluated statistically using the non-parametric JTK-Cycle test implemented in R [31].

## Results

### Locomotor activity

**Figure 1:**
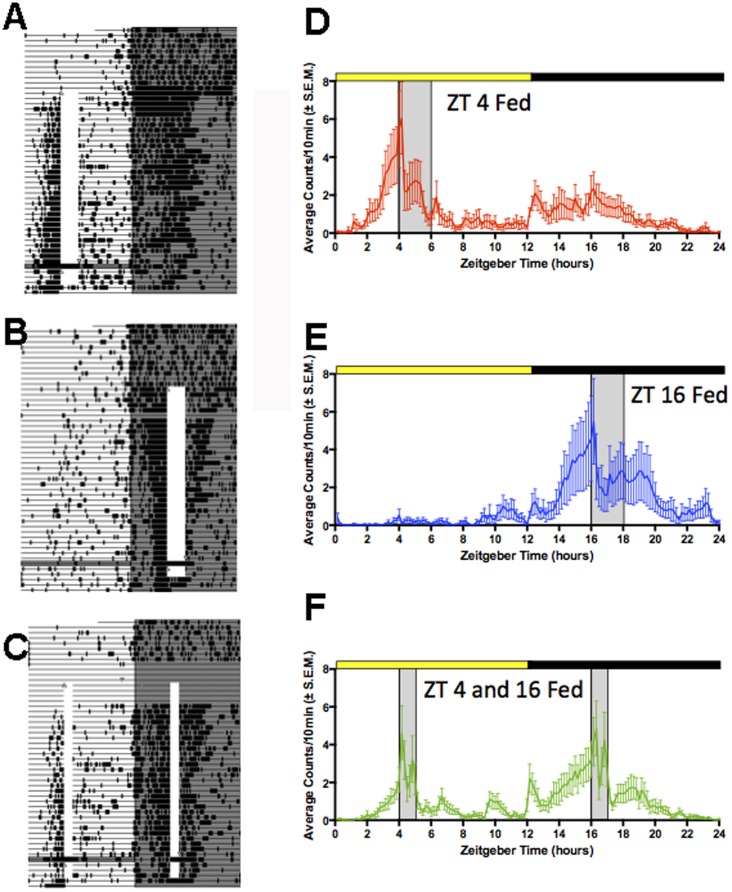
Locomotor activity recorded by motion sensors of rats restricted to 1 or 2 daily meals. A–C. Actograms from representative rats fed a 2 h daily meal at ZT4 (A) or ZT16 (B), or a 1 h meal at both times (C). Each line represents 24 h plotted from left to right in 10 min time bins. Time bins in which activity is registered are indicated by vertical deflections creating heavy bars when activity occurs across multiple consecutive bins. Mealtime is denoted by the opaque vertical bar. Lights-off is denoted by grey shading. D–F. Group mean average waveforms of activity during week 4 of restricted feeding, with mealtime at ZT4 (D), ZT16 (E) or at both times (F). Mealtimes are in grey shade. The daily 12 h light and dark periods are indicated by the heavy yellow and black bars, respectively.

Within a few days of scheduled feeding, all of the rats in each of the three groups showed a robust bout of activity in anticipation of daily feeding (Fig.1A–F). When food was omitted on day 33, the bout of food anticipatory activity persisted through the expected mealtimes before decreasing. Activity reappeared prior to mealtime on the next day, confirming that anticipation of mealtime, on one-meal and two-meal daily schedules, reflected a persisting circadian oscillation and not a one cycle hourglass clock timing intervals of 12 or 24 h between meals [7], [20].

### Adrenal clock genes: RT-PCR

**Figure 2:**
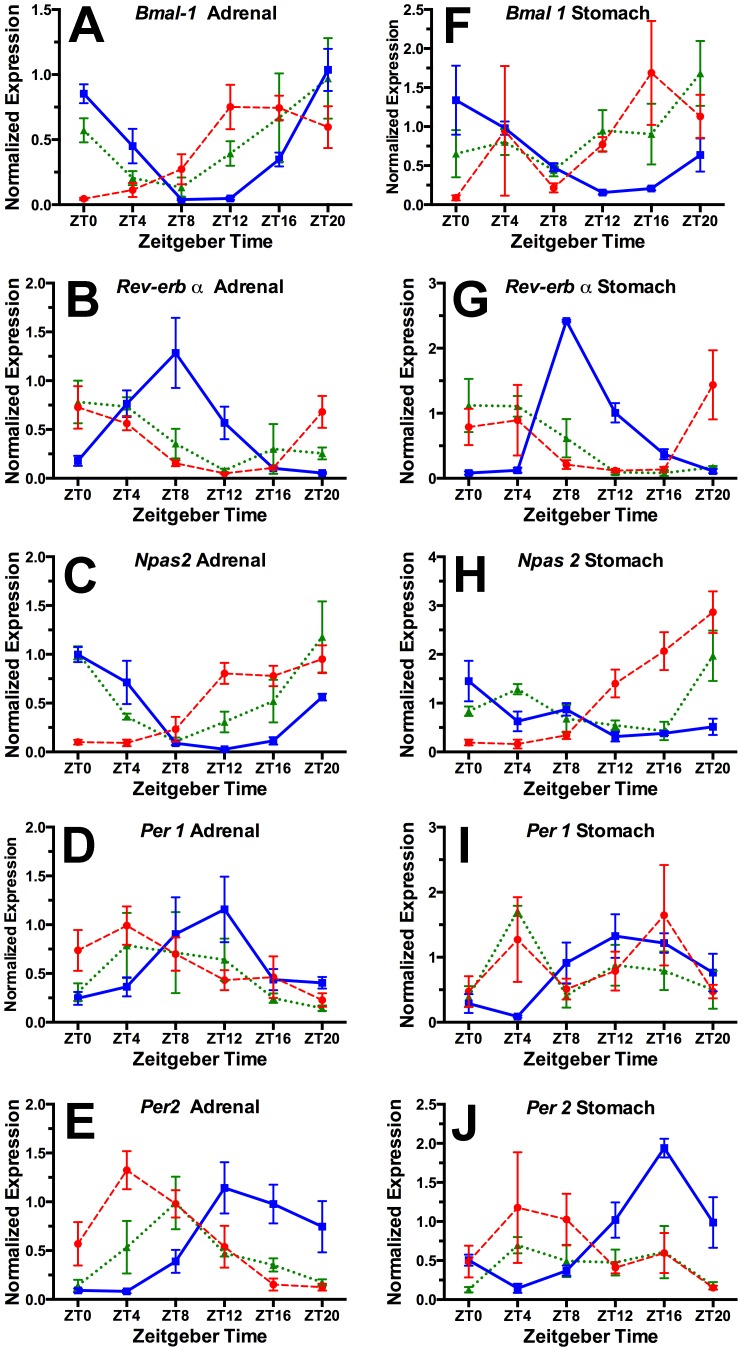
Normalized clock gene expression at 6 times of day from adrenal gland (A–E) and stomach (F–J) tissue collected from groups of rats fed for 2 h daily at ZT4 (red dashed curve) or ZT16 (blue solid curve), or for 1 h at both times (green dotted curve).

In the night (ZT16) fed rats, as expected, adrenal clock gene expression profiles (Fig. 2A–E) were very similar to those from ad-lib fed rats as described in previous studies [32]. This validates our assumption that a 2 h daily meal scheduled in the first half of the night would result in little or no shift in clock gene rhythms, and supports our decision to omit an ad-lib fed group. In this night-fed group, a significant 24 h rhythm was detected by JTK-cycle for each transcript (p<.002 or better; Table 1). The daily rhythms of *Bmal1* and *Npas2* peaked near the end of the night, *Per1* and *Per2* near the beginning of the night, and *Rev-erbα* in the second half of the day, which is in agreement with expression profiles observed in ad-lib fed animals [32].

**Table 1:** JTK-Cycle analysis of 24 h rhythmicity in peripheral clock gene expression and plasma hormones.

		ADRENAL GLAND			STOMACH		
gene	mealtime	ADJ.P	LAG	amp	ADJ.P	LAG	amp
**bmal1**	ZT16	0.000012	22	0.4999	0.000012	2	0.4822
	ZT4	0.000943	16	0.4292	0.019604	16	0.6371
	ZT4+16	0.002026	20	0.2510	ns	20	0.4363
**npas2**	ZT16	0.000006	1	0.3309	ns	2	0.1994
	ZT4	0.007951	17	0.5883	0.000943	18	0.8591
	ZT4+16	0.000104	22	0.3316	ns	4	0.2015
**per1**	ZT16	0.007951	12	0.1704	0.001392	16	0.6838
	ZT4	0.033993	6	0.3408	ns	15	0.0552
	ZT4+16	0.014667	8	0.2305	ns	9	0.1080
**per2**	ZT16	0.000104	16	0.5247	0.000000	18	0.4335
	ZT4	0.000167	6	0.5855	ns	9	0.2503
	ZT4+16	0.000628	10	0.1768	ns	10	0.1874
**reverb** ***α***	ZT16	0.000002	8	0.2956	0.000167	12	0.2524
	ZT4	0.000063	2	0.2828	ns	1	0.1676
	ZT4+16	0.004117	4	0.3535	0.001392	2	0.1400
		**CORTICOSTERONE**			**GHRELIN**		
	ZT16	0.00022	14	13.504	0.00005	18	287.93
	ZT4	ns	8	9.475	ns	12	139.93
	ZT4+16	0.03450	14	8.661	ns	12	39.38

Also as expected, rats fed in the day at ZT4 exhibited a marked shift in the daily profile of each clock gene, with peak expression approximately in antiphase to the profiles in the ZT16 group (Fig. 2). All of the clock gene transcripts exhibited a significant 24 h rhythm by JTK-Cycle analysis (Table 1). This confirms a previous report that the phase of the circadian clock in the adrenal gland is inverted by restricting food intake to the mid-light period, despite continued exposure to a LD cycle [33].

In contrast to the food anticipatory behavioral rhythm, clock gene rhythms in the adrenal gland in rats fed two daily meals retained a unimodal waveform. The profiles of *Rev-erbα, Per1* and *Per2* appear to have shifted, peaking either in phase with the ZT4 group (*Rev-erbα*, *Per1*) or midway between the ZT4 and ZT16 groups (*Per2*). The profiles of *Bmal1* and *Npas2* conform more closely to those of the ZT16 group, but also exhibit a peak phase midway between the peaks in the ZT4 and ZT16 one-meal groups (Table 1). These results indicate that the adrenal gland in rats fed twice daily at 12 h intervals, once in the day and once at night, continues to oscillate as a unitary circadian clock, but with a compromise phase position determined by both daily meals.

### Stomach clock genes: RT-PCR

The profiles of clock gene expression from stomach tissue in rats fed at ZT16 were very similar to the profiles from adrenal tissue, with all transcripts except *Npas2* showing a highly significant 24 h rhythm (Fig. 2F–J; Table 1). In the ZT4 condition the profiles for *Bmal1* and *Npas2* were markedly shifted, while the other transcript rhythms, although appearing shifted, were damped and not significant. The expression profiles in the 2-meal condition were more similar to the ZT4 condition, with only *Rev-erbα* exhibiting a significant 24 h rhythm. Again, the waveforms of the 5 clock genes in the 2-meal condition, taken together, are consistent with a unimodal, albeit damped circadian rhythm. By contrast with the adrenal clock, the stomach clock may be more strongly affected by a daytime meal in competition with a nighttime meal.

### Plasma corticosterone: Immunoassay

**Figure 3:**
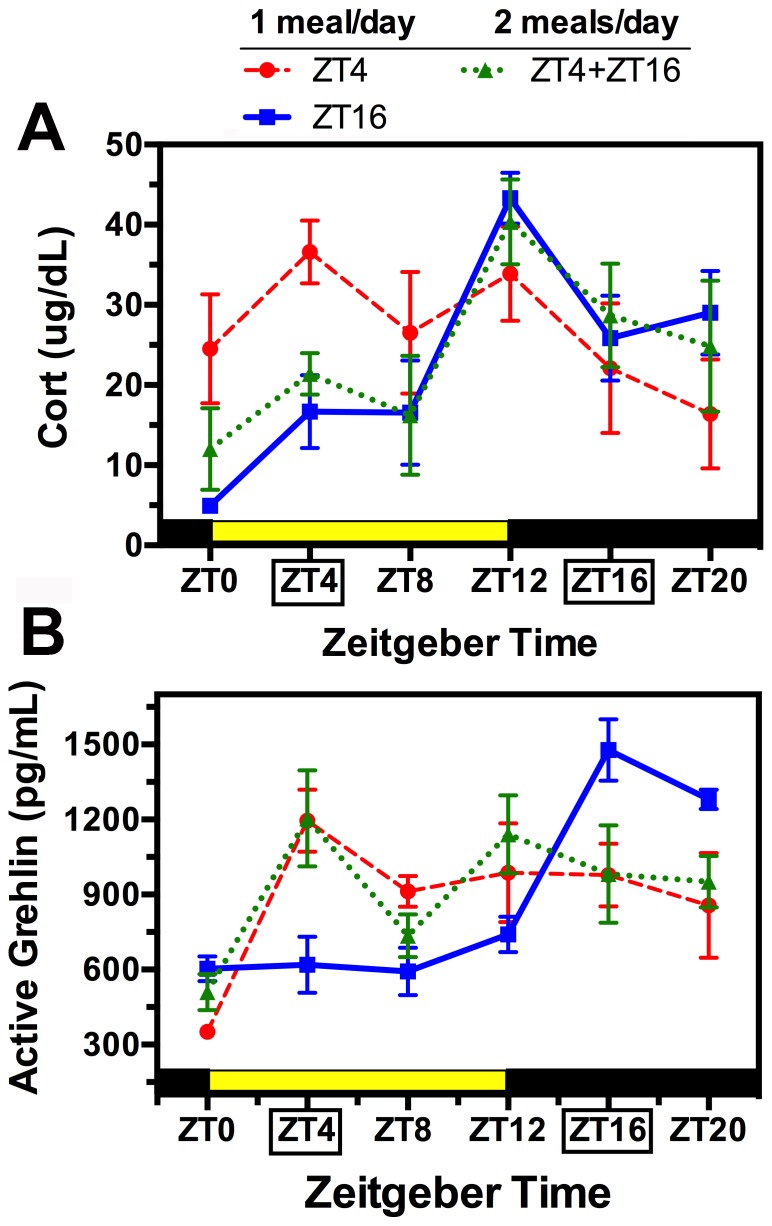
Group mean (± SEM) plasma corticosterone (A) and acyl-ghrelin (B) at 6 times of day from rats fed 2 h daily at ZT4 (red dashed curve) or ZT16 (blue solid curve), or for 1 h at both times (green dotted curve). The daily 12 h light and dark periods are indicated by the heavy yellow and black bars, respectively.

Previous studies show that in rats provided food ad-libitum, plasma corticosterone rises during the latter half of the daily rest phase (light period) and peaks at the beginning of the daily active phase (lights-off) [34], [35]. In the present study, rats fed at ZT16 showed a similar significant 24 h variation of plasma corticosterone, with a marked peak at ZT12 (4 h prior to mealtime; Fig. 3A; Table 1). In rats fed at ZT4, the daily variation in corticosterone was markedly altered and no longer significant. Corticosterone in this group was elevated at ZT0 and ZT4 compared to the ZT16 group, and slightly decreased at ZT12. This pattern conforms to previous reports that in daytime fed rats, corticosterone rises in anticipation of the daytime meal, but also shows a nocturnal peak, reflecting a continued influence of the LD entrained SCN pacemaker [36].

In the 2-meal condition, corticosterone exhibited a significant 24 h rhythm that conformed closely to the rhythm in the night-fed condition, with a peak at ZT12 and a slightly lower amplitude (Fig. 3a; Table 1). This result implies that feeding schedules influence the daily rhythm of plasma corticosterone via a single unitary clock mechanism, and that when two meals are in competition, the meal coinciding with the active phase is dominant. Lack of shifting of the corticosterone rhythm in the two-meal condition, despite partial shifting of the clock gene rhythms, suggests that feeding schedules can alter the phase relationship between the adrenal clock and an adrenal output hormone. This will need confirmation with higher resolution sampling.

### Plasma acyl-ghrelin Immunoassay

Previous studies show that in rats provided food ad-libitum, plasma acyl-ghrelin increases during the last hour of the light period, just prior to the onset of nocturnal feeding [36]. Consistent with this relationship between ghrelin secretion and nocturnal feeding, plasma acyl-ghrelin in the ZT16 fed rats exhibited a 3-fold 24 h rhythm with a peak at the expected mealtime (Fig. 3B; Table 1). In rats fed at ZT4, plasma acyl-ghrelin exhibited a lower amplitude rhythm that also peaked at the expected mealtime, and remained elevated for the remainder of the day (no food was provided). In the 2-meal condition, plasma acyl-ghrelin levels also peaked at ZT4, and looked similar to the day-fed group at other times of day, but a significant 24 h rhythm was not detected by JTK_Cycle. Thus, the daily rhythms of corticosterone and acyl-ghrelin were affected differently by the 2-meal schedule, with the corticosterone rhythm controlled predominantly by the nighttime meal, and the ghrelin rhythm predominantly by the daytime meal.

### Suprachiasmatic nucleus (SCN): *Bmal1* and *Per1* ISH

**Figure 4:**
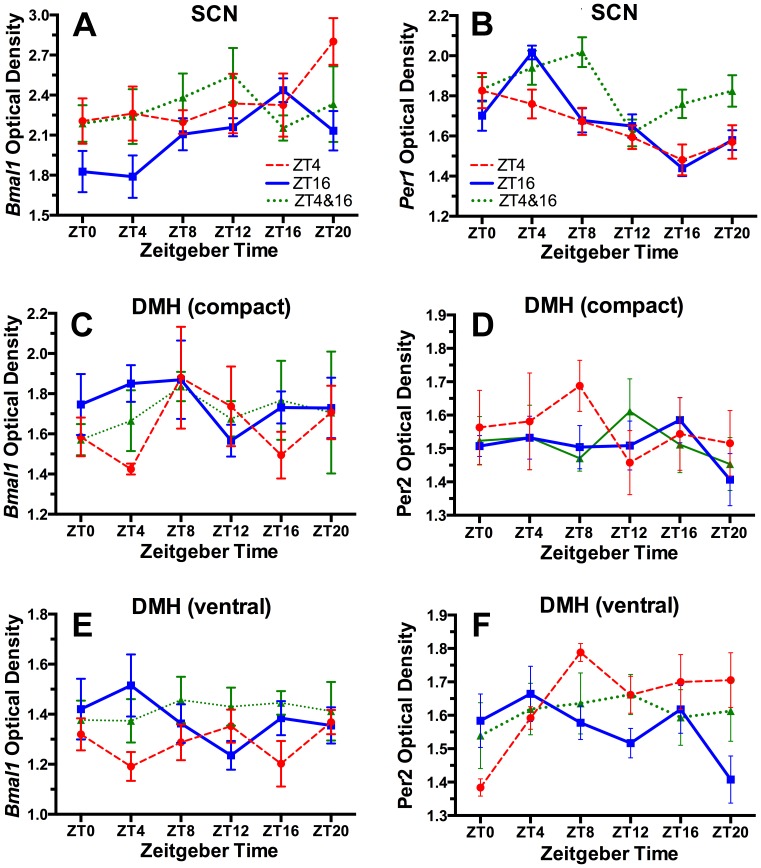
Group mean (± SEM) optical densities representing *Bmal1* and *Per1* mRNA from the SCN (A–B) and dorsomedial hypothalamus (DMH, compact and ventral regions, C–F) in rats fed 2 h daily at ZT4 (red dashed curve) or ZT16 (blue solid curve), or for 1 h at both times (green dotted curve).

Clock gene rhythms in the SCN, assessed by ISH, ICC or bioluminescent reporters, are generally assumed to be unperturbed by daytime restricted feeding schedules in rats and mice entrained to LD cycles, but shifts were reported in 1 of 3 studies that quantified *Bmal1* expression [37], and in 4 of 10 studies that quantified *Per1* mRNA or PER1 protein [9], [37], [38], [39]. Using ISH, we observed a significant rhythm of *Bmal1* expression in the SCN of rats fed at ZT16 (Table 2), with peak expression at ZT16, but a marked attenuation of this rhythm in both the ZT4 and the 2-meal groups (Fig. 4A), primarily due to increased expression in the day relative to the night-fed group. *Per1* also exhibited a significant rhythm in the ZT16 condition, with a peak identified at ZT6 by JTK-Cycle, in antiphase with *Bmal1*. In rats fed at ZT4, *Per1* a significant 24 h rhythm was evident, although the amplitude was reduced the peak identified at ZT4 (Fig. 4B). The 2-meal group showed elevated *Per1* expression at night, and a damped, non-significant 24 h rhythm. These results indicate that daytime and 2-meal schedules can alter clock gene expression in the SCN. The reduced expression of *Per1* and increased expression of *Bmal1* at mealtime in rats fed at ZT4 is consistent with other evidence that *Per1*, in nocturnal rodents, can be acutely suppressed by behavioral activation in the day [40].

**Table 2:** JTK-Cycle analysis of 24 h rhythmicity in clock gene expression in brain regions of interest.

region & gene	Meal time	ADJ.P	phase	amp	region & gene	Meal time	ADJ.P	phase	amp
**SCN**	ZT16	0.001	16	0. 230	**SCN**	ZT16	0.000	6	0.127
**Bmal1**	ZT4	ns	20	0.136	**Per1**	ZT4	0.016	4	0.107
	ZT4+16	ns	12	0.106		ZT4+16	ns	4	0.136
									
**vDMH**	ZT16	ns	17	0.009	**vDMH**	ZT16	ns	8	0.023
**Bmal1**	ZT4	ns	2	0.053	**Per2**	ZT4	0.039	14	0.125
	ZT4+16	ns	15	0.034		ZT4+16	ns	11	0.040
									
**cDMH**	ZT16	ns	4	0.165	**cDMH**	ZT16	ns	9	0.015
**Bmal1**	ZT4	ns	22	0.091	**Per2**	ZT4	ns	5	0.100
	ZT4+16	ns	7	0.011		ZT4+16	ns	11	0.006
									
**OB**	ZT16	0.000	10	0.377	**NAc core**	ZT16	0.001	12	0.132
**Bmal1**	ZT4	0.000	4	0.409	**Bmal1**	ZT4	0.000	4	0.072
	ZT4+16	ns	6	0.233		ZT4+16	ns	13	0.008
									
**dStr**	ZT16	0.004	0	0.258	**NAc shell**	ZT16	0.011	12	0.130
**Bmal1**	ZT4	ns	5	0.028	**Bmal1**	ZT4	0.001	4	0.124
	ZT4+16	ns	20	0.057		ZT4+16	ns	8	0.049
									
**Cerebellum**	ZT16	0.001	4	0.400	**Arcuate**	ZT16	0.039	22	0.165
**Bmal1**	ZT4	ns	11	0.043	**Bmal1**	ZT4	ns	4	0.091
	ZT4+16	0.021	4	0.240		ZT4+16	ns	7	0.011

### Dorsomedial Hypothalamus (DMH): *Bmal1* and *Per2* ISH

One lesion study implicated the DMH as critical for food anticipatory rhythms [41]. Subsequent studies showed that this region does not generate the food anticipation rhythm, but may facilitate the expression of daytime activity by suppressing sleep-promoting output from the SCN pacemaker prior to mealtime [42]–[46]. Although the DMH was claimed to be the only area in which *Per2* expression was induced to oscillate by daytime feeding [47], other studies have reported food-shifted rhythms of clock gene expression in numerous other brain regions [14, see references below]. Only 1 study has reported on *Bmal1* expression in the DMH of food restricted rats, and in that study no significant rhythm was observed [37]. We obtained the same result, with no significant rhythm of *Bmal1* expression evident in either the compact or ventral DMH, in any of the three restricted feeding conditions (Fig. 4C–F), using JTK-Cycle (Table 2) and 1-way ANOVA (data not shown). *Bmal1* was similarly arrhythmic in the compact DMH, in all three feeding conditions, and in the ventral DMH in the ZT16 and 2-meal groups. *Per2* expression exhibited a significant rhythm only in the ventral DMH in rats fed at ZT4, with a peak identified by JTK-Cycle at ZT14.

### Olfactory bulb (OB): *Bmal1* ISH

**Figure 5:**
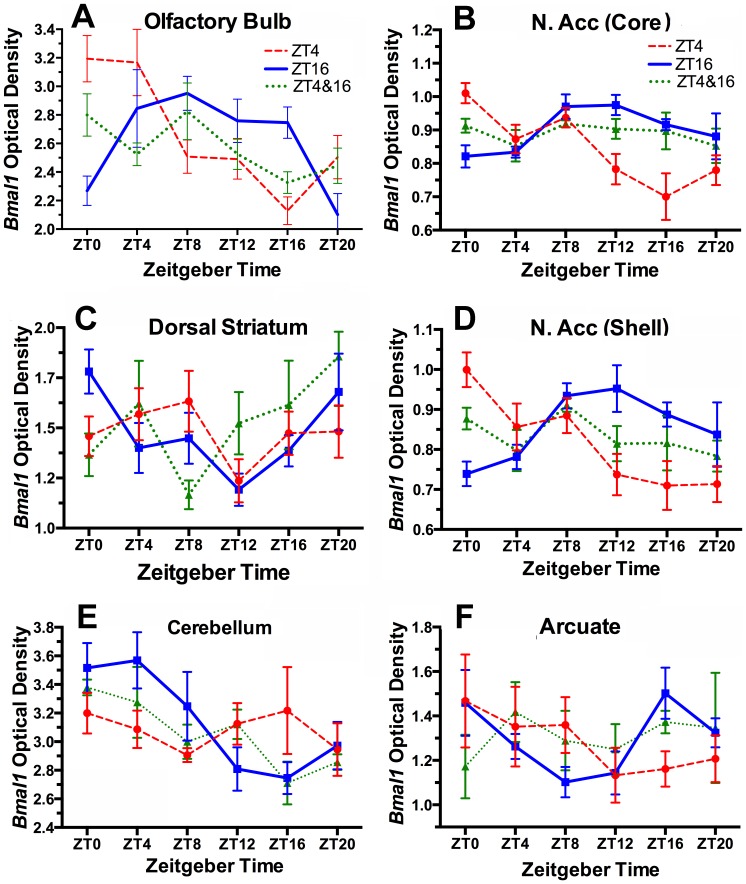
Group mean (± SEM) optical densities representing *Bmal1* mRNA from rats fed 2 h daily at ZT4 (red dashed curve) or ZT16 (blue solid curve), or for 1 h at both times (green dotted curve). A. Olfactory bulb, B. nucleus accumbens core, C. dorsal striatum, ventrolateral quadrant, D. nucleus accumbens shell, E. cerebellum, F. Arcuate nucleus.

In ad-lib fed rats and mice, the OB exhibits circadian oscillations of *Per1* and *Per2* that persist in vivo after SCN-ablation, and in long term tissue explants [48], [49]. Recent work in rats [50] and rabbit pups [51] has shown that *Per1* and *Per2* rhythms in the OB are entrainable by scheduled feeding. We observed a significant circadian oscillation of *Bmal1* expression in the OB that peaked at ZT10 in the night-fed group, and at ZT4 in the day-fed group (Fig. 5A; Table 2). Notably, *Bmal1* expression did not exhibit a significant rhythm in the 2-meal group; at 5 of 6 time points, expression levels fell mid-way between those evident in the ZT4 and ZT16 groups. Assuming that circadian oscillations in the OB persist at the cellular level, this result could reflect either complete desynchrony within a population of oscillators, or uncoupling between two populations of OB clock cells, each entrained to a different meal.

### Nucleus Accumbens (NAc): *Bmal1* ISH

Rats exhibit daily rhythms of PER1 and PER2 protein in the NAc core and shell that peak at about ZT18 when food is available ad-libitum, and that are phase advanced by 6 h when food is restricted to the middle of the light period [52], [53]. Despite the importance of the NAc for processing of reward stimuli, combined ablation of the core and shell had no effect on food anticipatory running in rats [54]. We confirm and extend previous findings by showing that *Bmal1* mRNA also exhibits a circadian rhythm in both the core and the shell, with peak levels identified at ZT12 in rats fed at night, and at ZT4 in rats fed in the day (Fig. 5B,D). This rhythm was absent in the rats fed at both times, and expression levels assumed values roughly intermediate between the day-fed and night-fed groups. The NAc therefore, in each feeding condition, exhibited the same patterns of *Bmal1* expression as were observed in the OB.

### Dorsal striatum (dStr): *Bmal1* ISH

The dStr expresses circadian rhythms of *Per1* and *Per2* mRNA and protein that can be shifted by daytime feeding schedules [13], [47], [55], [56] and by dopaminergic agonists [57]. The dStr has also been proposed as the site of dopamine sensitive circuits involved in interval timing [58]. Dopamine expression limited to the dStr is sufficient for dopamine-deficient mice to anticipate a daytime meal, and dopamine D1 receptor knockouts severely diminish food anticipatory running and general activity [59]. We quantified *Bmal1* expression in dorsomedial, ventromedial, dorsolateral and ventrolateral quadrants of the dStr. In the ZT16 fed rats, we observed a similar low amplitude oscillation in each of these quadrants, with greatest amplitude in the ventrolateral quadrant (Fig. 5C). JTK_Cycle identified a significant 24 h rhythm with a peak at ZT0 (Table 2). By contrast with the night-fed group, the day-fed rats exhibited an increasing trend in *Bmal1* expression during the light period but the two groups otherwise looked similar. Neither the day-fed nor the 2-meal rats exhibited a significant 24 h rhythm. Therefore, unlike the *Per* genes, which were shown to exhibit significant rhythms that were reset by daytime feeding compared to ad-lib food access [13], [46], [55], [56], *Bmal1* exhibits a lower amplitude rhythm that is disrupted by restricted feeding schedules that include a daytime meal.

### Cerebellum: *Bmal1* ISH

The mouse cerebellum exhibits circadian oscillations of *Per1, Per2* and *Rev-erbα* that are phase advanced by a restricted daytime feeding schedule [60], and at least weakly rhythmic in vitro [61]. Mutations that affect cerebellar and motor function can attenuate food anticipatory rhythms [60]. We observed a significant rhythm of *Bmal1* expression in the cerebellum of rats fed at ZT16, with a peak at ZT4 (Fig. 5E; Table 2). The *Bmal1* profile in the ZT4 group appears to be in antiphase with the ZT16 group profile (lower during the day, higher during the night), but a significant 24 h rhythm was not detected in this group. *Bmal1* expression did vary significantly with time of day in the 2-meal group, and although the mean values at each time point are not significantly different between groups, the values are intermediate between the 1-meal groups during the light period, and close to the 1-meal groups during the dark period. This relationship between the 2-meal condition and the 1-meal conditions is again broadly similar to that observed in the OB and NAc.

### Arcuate Nucleus: *Bmal1* ISH

The arcuate nucleus is an important integrative zone for peripheral metabolic signals (e.g., leptin, ghrelin, insulin) that regulate metabolism and ingestive behavior, and that can modulate the expression of food anticipatory rhythms [28], [62], [63], [64], [65]. The arcuate nucleus exhibits circadian oscillations of *Per* genes in vivo and in vitro [66], [67]. Shifting of circadian profiles of *Per1, Per2* and *Bmal1* mRNA or protein in response to a restricted daytime feeding schedule has been reported in mice and rats [13], [37], [45], [53]. We observed a significant, albeit low amplitude rhythm of *Bmal1* expression in the arcuate nucleus in the ZT16 fed rats, but no significant rhythm in either the ZT4 group, or the 2-meal group (Fig. 5F). The profile in the day-fed group looks approximately anti-phasic relative to the night fed group (higher in the day, lower at night), while the profile in the 2-meal group is again relatively flat and at intermediate levels.

### Regions of interest that did not exhibit significant rhythms of *Bmal1* expression

*Bmal1* expression was also evaluated in the basolateral and central amygdala, lateral and medial habenula, paraventricular nucleus of the thalamus, barrel cortex and piriform cortex, as one or more studies have reported significant rhythms of *Per1* or *Per2* in these areas under ad-lib feeding conditions [13], [14], [52], [66]. None of these studies quantified *Bmal1* expression under restricted feeding conditions. No significant 24 h rhythm was detected in any of these regions, in any of the three feeding conditions, by JTK_Cycle analysis (S2 Table; S3 Figure).

## Discussion

To gain insight into the location and organization of food-entrainable oscillators controlling behavior, we examined expression of circadian clock genes in multiple central and peripheral tissues in rats anticipating one or two daily mealtimes. We postulate two models by which food-entrainable oscillators could be coupled to behavior. In one model, food-entrainable oscillators entrain to one daily meal, and drive an anticipatory behavioral rhythm to that meal alone. Anticipation of two daily meals [and potentially more; 16] would therefore involve at least two food-entrainable oscillators (or populations of oscillators) that can uncouple and entrain independently to different feeding times. An alternative model invokes the concept of the continuously consulted clock. According to this concept, circadian oscillators emit time signals that permit discrimination of circadian phase (and time of day, if these oscillators are entrained to local time), and memory of mealtime if meals occur at predictable circadian phases. Anticipation of two (or more) mealtimes would therefore reflect discrimination and memory for time, as represented by a single clock (presumably comprised of multiple, coupled clock cells). Given the persistence of food anticipatory rhythms in SCN ablated rodents, a continuously consulted clock signaling mealtimes must be elsewhere in the brain or body [68]. The results of our study indicate that circadian oscillators in peripheral tissues (specifically, the stomach and adrenal gland) can entrain to a single daytime or a nighttime meal, and assume a compromise phase position under the joint influence of 2 daily meals. Circadian oscillators in multiple brain regions examined also synchronized to a single daytime or nighttime meal, but exhibited patterns of expression during 2-meal schedules that suggest two populations of oscillators cycling in antiphase, to be discussed further below.

Peripheral signals, most notably the gastric hormone acyl-ghrelin, have been proposed to participate in food anticipatory activity rhythms in one or more of three possible ways, 1. as internal zeitgebers that entrain central oscillators driving food anticipatory activity, 2. as the output of entrained oscillators that drive anticipatory activity, or 3. as modulators of the amount of anticipatory activity. Ghrelin does not appear to be necessary for food anticipatory activity [69], [70], [71] although anticipatory activity rhythms may exhibit a reduced peak level or duration in receptor knockouts, supporting a modulatory role [28], [63], [65]. It was previously shown that circadian oscillations of the clock gene *Per1* in liver and stomach entrain to the nighttime meal in rats robustly anticipating a daytime meal at ZT4 and a nighttime meal at ZT16 [72], although the liver clock does shift if the daytime meal is much larger or precedes the nighttime by 8 h [73]. We also observed dissociations between peripheral clock gene rhythms and food anticipation, and have extended this to hormonal outputs of the peripheral clocks. Clock gene rhythms in the stomach and adrenal were inverted in daytime fed rats compared to nighttime fed rats, and assumed a unimodal, intermediate phase position in the 2-meal fed rats. The rhythm of plasma ghrelin peaked at the expected mealtime in both 1 meal conditions, but at only the daytime meal in the 2-meal condition. Conversely, plasma CORT peaked only prior to the nighttime meal in the 2-meal condition. The timing of the gastric and adrenal clocks, and associated hormonal outputs, under the 2-meal condition indicates that these signals cannot be responsible for timing food anticipatory activity under 2-meal schedules. The results do not rule out a role for ghrelin as a modulatory factor affecting the amount of food anticipatory activity.

Previous work, using ISH, immunolabeling or bioluminescent reporters, has described daily rhythms of clock gene expression in numerous brain regions in rats and mice [66], [67]. Where this has been tested, rhythms in most of these areas are markedly shifted by a daytime feeding schedule [14]. Most of these studies measured *Per1* or *Per2*. Our work extends these findings by quantifying *Bmal1* expression, supplemented in two regions with ISH for *Per1* or *Per2. Bmal1* is thought to play a critical role as transcriptional activator of *Per* and *Cry* genes that comprise the negative feedback loop at the core of the mammalian circadian clock [1]-[4]. Although mice lacking *Bmal1* can still anticipate a daily meal, the *Bmal1*-independent food anticipatory activity rhythm loses circadian constraints [23]. We examined *Bmal1* expression in 14 brain regions, 11 of which have previously been reported to exhibit *Per1* or *Per2* rhythms reset by a daytime feeding schedule [13], [14], [52], [66]. Seven of these regions exhibited a significant rhythm of *Bmal1* expression in rats fed once daily at night. In 5 regions (OB, NAc, dStr, cerebellum and arcuate nucleus) the rhythm was approximately inverted in rats fed once daily at ZT4. In 2 regions (SCN and DMH), the rhythm was disrupted and not significant in rats fed at ZT4. In the other 7 brain regions examined (basolateral and central amygdala, barrel cortex, piriform cortex, lateral and medial habenula, and paraventricular nucleus of the thalamus), *Bmal1* expression did not exhibit a significant rhythm in any of the three feeding conditions. Daily rhythms of *Per1* or *Per2* expression that have been reported in these areas [13], [14], [53], [66] may reflect the operation of a *Bmal1*-independent oscillator, or may be directly driven by cellular inputs related to feeding or activity. Evidence for this has been reported in conditional *Bmal1* knockouts directed at the liver. In these animals *Bmal1* was not rhythmically expressed in the liver yet *Per2* remained rhythmic [74].

Although the SCN, in rodents entrained to LD cycles, are generally assumed to be resistant to shifting by daytime feeding schedules, several studies, one of which measured *Bmal1* expression [36], have reported some modification of phase. We observed an attenuation in the amplitude of the SCN *Bmal1* profile in rats fed at ZT4, or at both ZT4 and ZT16. In both conditions, *Bmal1* expression was increased in the day and decreased in the middle of the night. In the ZT4 condition, *Per1* expression was decreased at mealtime, the time of peak expression in the ZT16 group. *Per1* expression in the Syrian hamster SCN in the middle of the light period is suppressed by stimulated arousal [40]. cFos expression is also attenuated [75], and these effects can be induced by neurotransmitters utilized by the intergeniculate leaflet and median raphe, two SCN inputs thought to mediate nonphotic effects on the SCN pacemaker [76]. In rats and mice, daytime food anticipatory activity is also associated with suppression of SCN *cFos* [45], [77]. The reduced expression of *Per1* at mealtime in our ZT4 fed rats thus could represent a feedback effect of behavior on SCN clock cells.

Alternatively, suppression of SCN *Per1* at ZT4 in our rats and SCN *cFos* as reported in other studies could be mediated by inputs from the DMH, a region that is activated both before and during a scheduled daytime meal, and that has presumptively inhibitory projections to the SCN [40], [44], [45], [78]. Although the DMH has been proposed to function as a food-entrained oscillator, the supporting evidence was derived from quantification of *Per1* and *Per2* expression alone [47]. Only one previous study quantified *Bmal1* expression in the DMH, and that study reported no significant rhythm in ad-lib fed or mid-day fed rats [37]. We also failed to observe a significant rhythm of *Bmal1* expression in any of three feeding conditions. The only significant rhythm that we did observe was *Per2* expression in the ventral DMH in rats fed at ZT4. This confirms that *Per2* does exhibit daily variation in day-fed rats. If the lack of significant *Bmal1* rhythmicity in this group is real (i.e., not due to a lack of sensitivity of the method to low amplitude rhythmicity), then the presence of rhythmicity in *Per2* expression suggests that the rhythm may be driven directly by signals related to feeding, and not due to *Bmal1*.

The OB and NAc core and shell all exhibited relatively robust daily rhythms of *Bmal1* in rats fed at night, and inversion of these rhythms in rats fed in the day. Importantly, in rats fed at both times, the rhythm of *Bmal1* was absent, and expression levels at most of the time points fell between the levels in the ZT4 and ZT16 one-meal groups. This absence of rhythmicity at the tissue level in the two-meal condition, despite robust anticipatory activity at the behavioral level, is suggestive of a 2-oscillator model in which food-entrainable oscillators uncouple and reorganize into separate populations that entrain to one or the other meal. By oscillating in antiphase with each other, non-rhythmic expression at intermediate levels would be the expected outcome. It is also possible that the absence of rhythmicity at the tissue level is due to the loss of rhythmicity at the cellular level, but there is no precedence for this in the published literature on wild-type rodents exposed to non-circadian lighting or feeding schedules. Even mice rendered behaviorally arrhythmic by exposure to constant bright light exhibit circadian oscillations at the cellular level [79]. Absence of rhythmicity at the tissue level could also be due to averaging across a group of animals in which equal numbers were entrained to one meal or the other, but there is again no precedence for this type of differential binary response to two-meal schedules, at the behavioral or tissue level. To further substantiate a 2-oscillator interpretation of the OB and NAc *Bmal1* results, recordings of clock gene rhythms at the single cell level are needed, e.g., using real time bioluminescent reporters.

Three other brain regions (dStr, cerebellum, arcuate nucleus) exhibited a significant rhythm of *Bmal1* expression in the ZT16 one-meal condition, but not in the day fed or two-meal conditions. Nonetheless, the relationships among the 3 profiles for each region are broadly similar to the relationships observed in the OB and NAc. *Bmal1* tended to be high at time points in the ZT4 condition when it was low in the ZT16 condition, and vice versa, while in the 2-meal condition, *Bmal1* expression levels were approximately intermediate.

In conclusion, this study used 1 and 2-meal feeding schedules to probe the organization of the circadian system that mediates food anticipatory rhythms in rats. The results help build a roadmap toward a deeper understanding of how circadian time is represented in the brain and body. Unimodal rhythms in phase with one or the other of two daily meals, or at a compromise phase, would constitute definitive evidence for one oscillator models. Circadian clocks in the periphery appear to conform to a single oscillator model. The more complex patterns evident in most of the brain regions examined are more consistent with a 2-oscillator model. This will need to be investigated further using repeated measures sampling of clock cycling in real time with single cell resolution.

## Supporting Information

S1 Figure **Schematic of experimental groups and data collection intervals.**
(TIF)

S2 Figure **Nissl stained coronal sections (from Allen brain atlas) indicating the approximate locations of brain regions of interest for in situ hybridization, and representative ISH examples.**
(TIF)

S3 Figure **Group mean waveforms of** ***Bmal1*** **expression in brain regions that did not exhibit significant 24 h rhythms in any of the three feeding groups.**
(TIF)

S1 Table **RT PCR Primers.**
(DOCX)

S2 Table **JTK-Cycle analysis of 24 h rhythmicity in clock gene expression in brain regions of interest.**
(DOCX)
