# Correction: Circadian Mechanisms of Food Anticipatory Rhythms in Rats Fed Once or Twice Daily: Clock Gene and Endocrine Correlates

**DOI:** 10.1371/journal.pone.0120223

**Published:** 2015-03-23

**Authors:** The PLOS ONE Staff

The ninth author’s name is spelled incorrectly. The correct name is: Seung Hwan Chung.
