# Supplementary figures and images for: Circadian Mechanisms of Food Anticipatory Rhythms in Rats Fed Once or Twice Daily: Clock Gene and Endocrine Correlates
Source: PLoS One. 2014 Dec 11;9(12):e112451. doi: 10.1371/journal.pone.0112451 (PMC4263600; doi:10.1371/journal.pone.0112451)

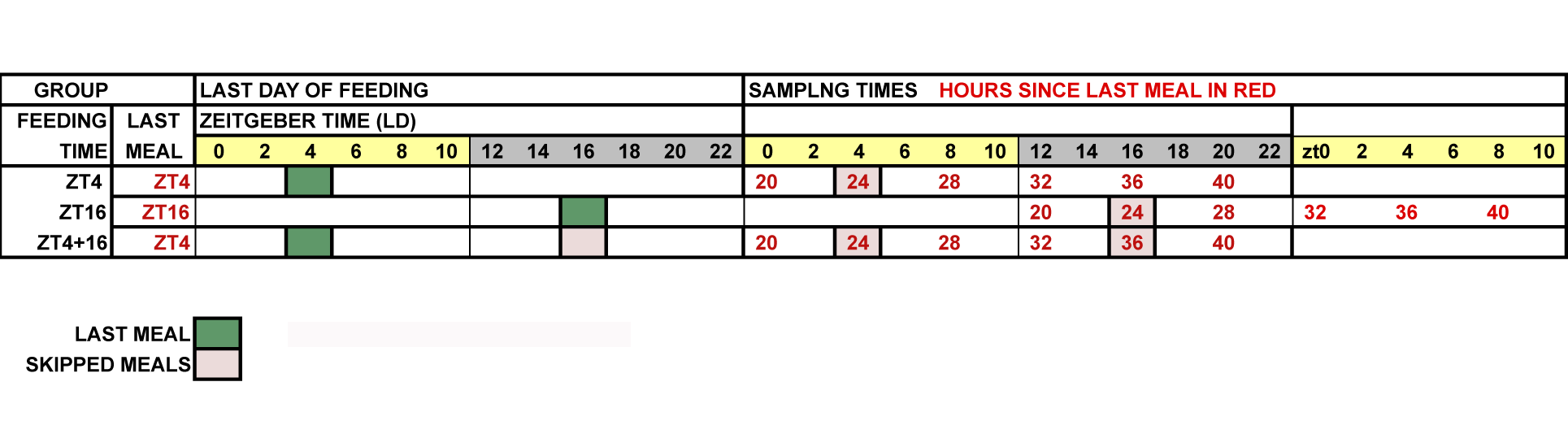

Supplement: S1 Figure — Schematic of experimental groups and data collection intervals. (TIF) [file pone.0112451.s001.tif]

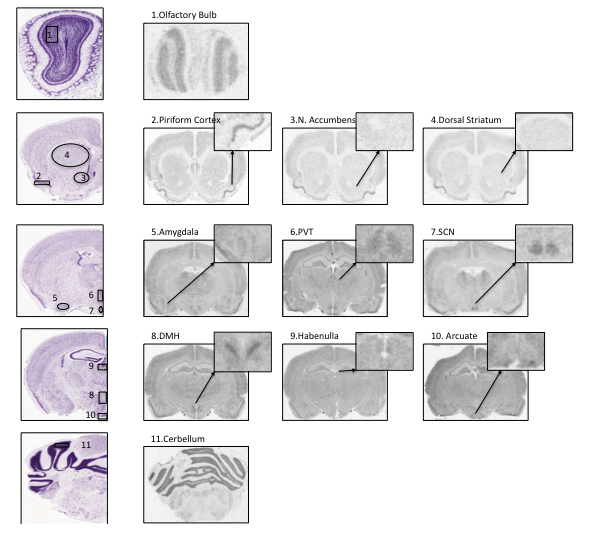

Supplement: S2 Figure — Nissl stained coronal sections (from Allen brain atlas) indicating the approximate locations of brain regions of interest for in situ hybridization, and representative ISH examples. (TIF) [file pone.0112451.s002.tif]

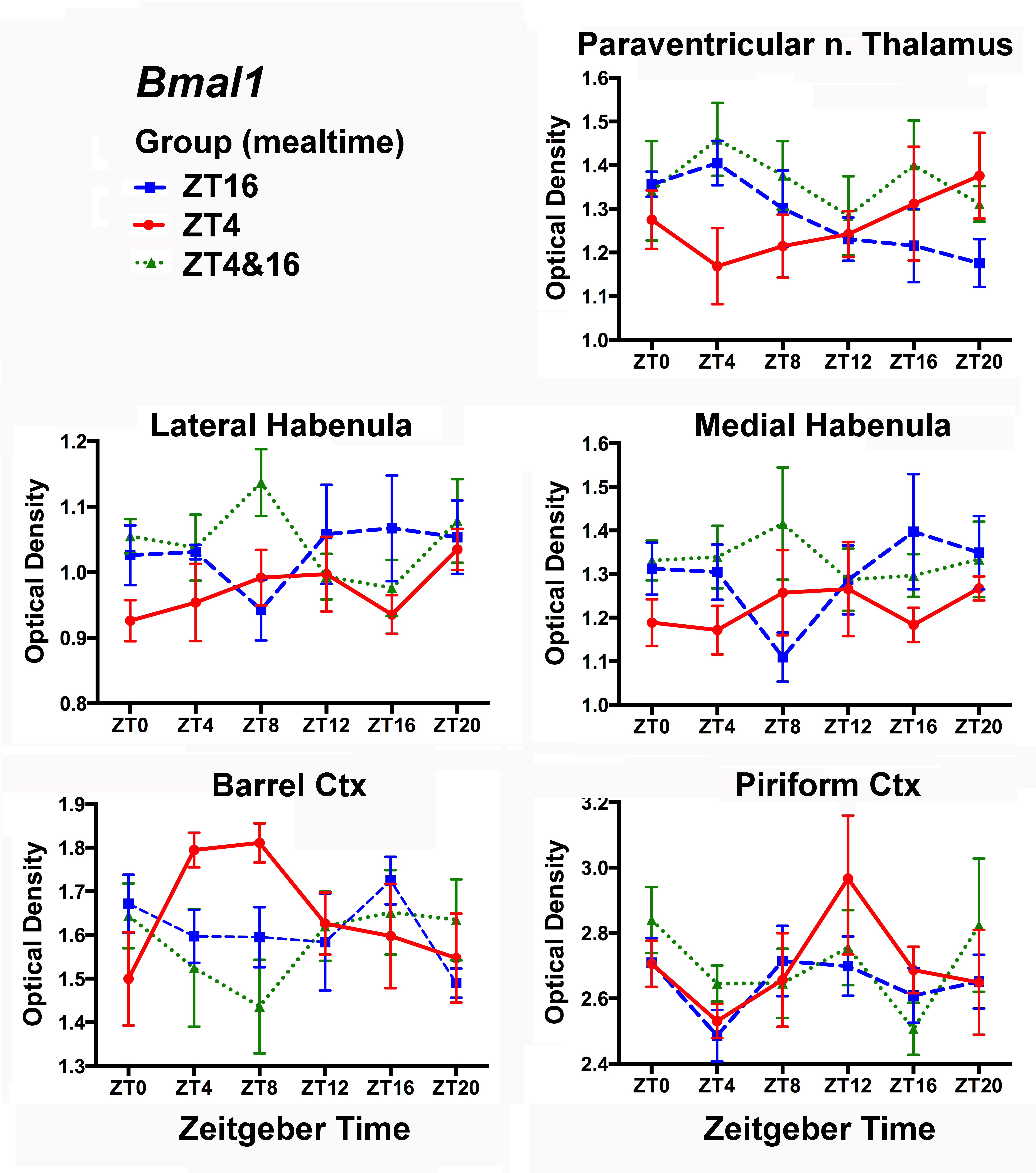

Supplement: S3 Figure — Group mean waveforms of Bmal1 expression in brain regions that did not exhibit significant 24 h rhythms in any of the three feeding groups. (TIF) [file pone.0112451.s003.tif]
