# Supplementary material for: Circadian Mechanisms of Food Anticipatory Rhythms in Rats Fed Once or Twice Daily: Clock Gene and Endocrine Correlates
Source: PLoS One. 2014 Dec 11;9(12):e112451. doi: 10.1371/journal.pone.0112451 (PMC4263600; doi:10.1371/journal.pone.0112451)
Supplement: S1 Table — RT PCR Primers. (DOCX) [file pone.0112451.s004.docx]

**Supplementary Table S1 RT PCR Primers**

| Gene | Sequence(5’-3’) |
| --- | --- |
| *Per1* | (F) ACACCCAGAAGGAAGAGCAA  (R) GCGAGAACGCTTTGCTTTAG |
| *Per2* | (F) CACCCTGAAAAGAAAGTGCGA  (R) CAACGCCAAGGAGCTCAAGT |
| *Rev-erb α* | (F) ACAGCTGACACCACCCAGATC  (R) CATGGGCATAGGTGAAGATTTCT |
| *Npas2* | (F) ATCTGTGACATCCAGCAGGA  (R) GGACACATAGATGATGCTGC |
| *Bmal-1* | (F) CTTGCGGAATGTCACAGGCA  (R) ACACCAGTGTTGGTTGAGAC |
| *Gapdh* | (F) ATGTCGTGGAGTCTACTGGC  (R) AGGATGCATTGCTGACAATC |
