# Supplementary material for: Circadian Mechanisms of Food Anticipatory Rhythms in Rats Fed Once or Twice Daily: Clock Gene and Endocrine Correlates
Source: PLoS One. 2014 Dec 11;9(12):e112451. doi: 10.1371/journal.pone.0112451 (PMC4263600; doi:10.1371/journal.pone.0112451)
Supplement: S2 Table — JTK-Cycle analysis of 24 h rhythmicity in clock gene expression in brain regions of interest. (DOCX) [file pone.0112451.s005.docx]

**Supplementary Table S3. JTK-Cycle analysis for 24h rhythmicity in clock gene expression in brain regions of interest.**

**For graphs, see Supplementary Figure S3.**

| **ROI and** | **Meal** | **Adjusted** |  |  |  | **ROI and** | **Meal** | **Adjusted** |  |  |
| --- | --- | --- | --- | --- | --- | --- | --- | --- | --- | --- |
| **gene** | **time** | **P value** | **phase** | **amp** |  | **gene** | **time** | **P value** | **phase** | **amp** |
| **Lateral** | ZT16 | 1.00 | 11 | 0.014 |  | **Medial** | ZT16 | 0.20 | 22 | 0.054 |
| **Habenula** | ZT4 | 1.00 | 17 | 0.000 |  | **Habenula** | ZT4 | 1.00 | 17 | 0.009 |
|  | ZT4+16 | 0.72 | 6 | 0.047 |  |  | ZT4+16 | 1.00 | 5 | 0.034 |
|  |  |  |  |  |  |  |  |  |  |  |
| **Barrel** | ZT16 | 1.00 | 13 | 0.000 |  | **Piriform** | ZT16 | 1.00 | 15 | 0.014 |
| **Cortex** | ZT4 | 0.10 | 8 | 0.037 |  | **Cortex** | ZT4 | 1.00 | 15 | 0.026 |
|  | ZT4+16 | 0.98 | 20 | 0.122 |  |  | ZT4+16 | 1.00 | 9 | 0.024 |
|  |  |  |  |  |  |  |  |  |  |  |
| **Amygdala** | ZT16 | 0.85 | 2 | 0.021 |  | **Para-** | ZT16 | 0.06 | 4 | 0.075 |
| **Basolateral** | ZT4 | 1.00 | 9 | 0.022 |  | **ventricular** | ZT4 | 1.00 | 13 | 0.003 |
| **ventral** | ZT4+16 | 1.00 | 11 | 0.025 |  | **thalamus** | ZT4+16 | 1.00 | 5 | 0.049 |
|  |  |  |  |  |  |  |  |  |  |  |
| **Amygdala** | ZT16 | 1.00 | 17 | 0.023 |  | **Amygdala** | ZT16 | 0.53 | 16 | 0.025 |
| **Basolateral** | ZT4 | 0.17 | 0 | 0.069 |  | **Basolateral** | ZT4 | 1.00 | 9 | 0.050 |
| **anterior** | ZT4+16 | 1.00 | 9 | 0.022 |  | **posterior** | ZT4+16 | 1.00 | 17 | 0.033 |
|  |  |  |  |  |  |  |  |  |  |  |
| **Amygdala** | ZT16 | 1.00 | 13 | 0.023 |  | **Amygdala** | ZT16 | 1.00 | 5 | 0.012 |
| **central** | ZT4 | 1.00 | 9 | 0.025 |  | **central** | ZT4 | 1.00 | 9 | 0.008 |
| **lateral** | ZT4+16 | 1.00 | 7 | 0.012 |  | **medial** | ZT4+16 | 0.09 | 14 | 0.054 |
